# *Vibrio harveyi*: a serious pathogen of fish and invertebrates in mariculture

**DOI:** 10.1007/s42995-020-00037-z

**Published:** 2020-04-03

**Authors:** Xiao-Hua Zhang, Xinxin He, Brian Austin

**Affiliations:** 1 MOE Key Laboratory of Marine Genetics and Breeding, College of Marine Life Sciences, Ocean University of China, Qingdao, 266003 China; 2 Laboratory for Marine Ecology and Environmental Science, Qingdao National Laboratory for Marine Science and Technology, Qingdao, 266237 China; 3 Frontiers Science Center for Deep Ocean Multispheres and Earth System, Ocean University of China, Qingdao, 266100 China; 4 Institute of Aquaculture, University of Stirling, Stirling, FK9 4LA Scotland, UK

**Keywords:** *Vibrio harveyi*, Pathogen, Fish, Invertebrates, Aquaculture

## Abstract

*Vibrio harveyi*, which belongs to family *Vibrionaceae* of class *Gammaproteobacteria*, includes the species *V. carchariae* and *V. trachuri* as its junior synonyms. The organism is a well-recognized and serious bacterial pathogen of marine fish and invertebrates, including penaeid shrimp, in aquaculture. Diseased fish may exhibit a range of lesions, including eye lesions/blindness, gastro-enteritis, muscle necrosis, skin ulcers, and tail rot disease. In shrimp, *V. harveyi* is regarded as the etiological agent of luminous vibriosis in which affected animals glow in the dark. There is a second condition of shrimp known as *Bolitas negricans* where the digestive tract is filled with spheres of sloughed-off tissue. It is recognized that the pathogenicity mechanisms of *V. harveyi* may be different in fish and penaeid shrimp. In shrimp, the pathogenicity mechanisms involved the endotoxin lipopolysaccharide, and extracellular proteases, and interaction with bacteriophages. In fish, the pathogenicity mechanisms involved extracellular hemolysin (encoded by duplicate hemolysin genes), which was identified as a phospholipase B and could inactivate fish cells by apoptosis, via the caspase activation pathway. *V. harveyi* may enter the so-called viable but nonculturable (VBNC) state, and resuscitation of the VBNC cells may be an important reason for vibriosis outbreaks in aquaculture. Disease control measures center on dietary supplements (including probiotics), nonspecific immunostimulants, and vaccines and to a lesser extent antibiotics and other antimicrobial compounds.

## Introduction

*Vibrio harveyi,* which occurs naturally in marine habitats (Firmino et al. 2019; Zhang et al. 2018), has developed into a significant pathogen of wild and cultured marine fish and invertebrates (notably penaeid shrimp), especially in warm waters of Asia, southern Europe, and South America. However, *V. harveyi* is not always recovered as a pure culture from diseased animals. Instead, there is some evidence that the pathogen may be recovered in mixed microbial populations, with, for example, *V. chagassi* (Fabbro et al. 2011). Moreover, *V. alginolyticus*, *V. harveyi,* and *V. parahaemolyticus* were detected in diseased yellow croaker (*Pseudosciaena crocea*) in China; none of the three bacterial pathogens dominated (Liu et al. 2016). Similarly, *V. harveyi* was recovered with *V. alginolyticus* from diseased hybrid groupers (*Epinephelus polyphekadion* × *E. fuscoguttatus*) in Malaysia *(*Mohamad et al. 2019). Of course, it is uncertain whether the presence of two or more bacterial taxa from the same pathological material represents co-culture or the presence of secondary invaders or even chance contaminants. Unfortunately, this aspect of pathology is rarely investigated. This review strives to discuss the biology of *V. harveyi* particularly aspects which appertain to its role as a serious pathogen of mariculture.

## Taxonomy

**Fig. 1:**
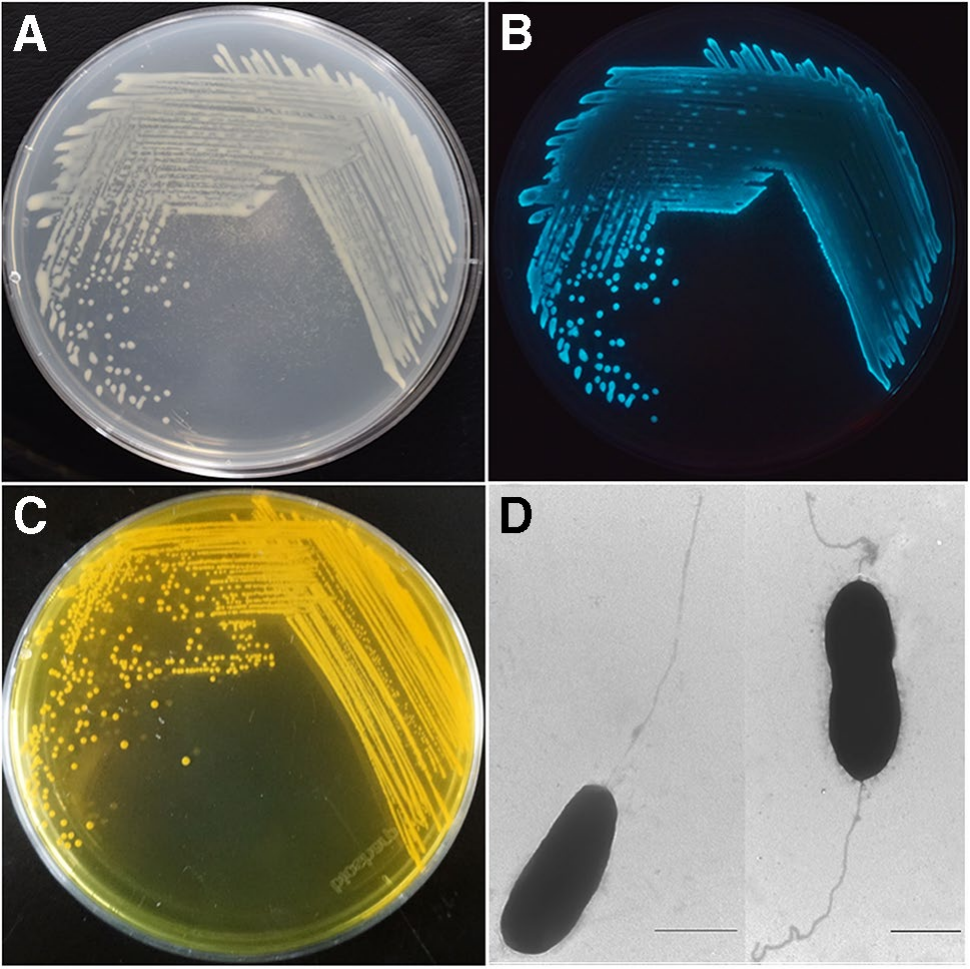
Morphology of *Vibrio harveyi* strains. **a** Growth of *V. harveyi* VIB 391 on marine agar 2216E; **b** luminescence of *V. harveyi* VIB 391; **c** growth of *V. harveyi* VIB 645 on TCBS agar; **d** transmission electron microscopy of VIB 645 cells obtained from marine broth culture. Scale = 1 μm

*Vibrio harveyi* is a member of the genus *Vibrio*, in the family *Vibrionaceae*, order *Vibrionales*, class *Gammaproteobacteria,* and phylum *Proteobacteria*. The classification of the organism has progressed from its initial name of *Achromobacter harveyi* (E. N. Harvey was a pioneer in the systematics of bioluminescence; Johnson and Shunk 1936) to *Lucibacterium harveyi* and *Beneckea harveyi* to the currently accepted name, *V. harveyi* (Farmer et al. 2005). Identification of isolates has progressed from phenotypic, serologic to genotypic or polyphasic, including the use of DNA:DNA hybridization when Ishimaru and Muroga (1997) confirmed that pathogenic isolates from milkfish (*Chanos chanos*) in Japan were definitely *V. harveyi.* A brief phenotypic description mentions that *V. harveyi* comprises Gram-negative, fermentative rod-shaped bacteria, which require for sodium chloride for growth and are motile by polar flagella (Fig. 1a, b). Growth occurs on TCBS (cholera agar = thiosulfate citrate bile salts sucrose agar) (Fig. 1c; Farmer et al. 2005). Some cultures are luminous (Fig. 1d). However, the phenotype may be changed by infection with the *Vibrio harveyi* myovirus like bacteriophage (VHML) (Vidgen et al. 2006). By 16S rRNA gene or whole-genome sequencing, *V. harveyi* is clearly a core species of *Vibrio* (Dorsch et al. 1992; Lin et al. 2018).

*Vibrio harveyi* was linked to *V. campbellii* by phenotyping and genotyping (Gomez-Gil et al. 2004). They noted that some isolates, which were labeled as *V. harveyi,* belonged in *V. campbellii.* However, multilocus sequence analysis confirmed the distinctness of *bona fide* isolates of *V. harveyi* and *V. campbellii,* and verified that both species are, indeed, separate entities (Thompson et al. 2007). Certainly, questions have been raised concerning the accuracy of identifying vibrios within the Harveyi clade. This problem may reflect on the associated studies discussed below (Hoffmann et al. 2012; Ke et al. 2017; Urbanczyk et al. 2013).

*Vibrio carchariae* was named after recovery of an isolate from a dead sandbar shark (*Carcharhinus plumbeus*) (Grimes et al. 1984) and lemon sharks (*Negraprion brevirostris*) (Colwell and Grimes 1984). However, detailed taxonomic examination, including the use of 16S rRNA gene sequencing (Gauger and Gomez-Chiarri 2002), led to the recognition that *V. carchariae* and *V. harveyi* are virtually identical, with the name of *harveyi* having precedence (Farmer and Hickman-Brenner 1992; Gauger and Gomez-Chiarri 2002; Pedersen et al. 1998). It is noteworthy that isolates identified as *V. carchariae* have been isolated from humans, who was bitten by sharks (Pavia et al. 1989).

The fish pathogen *V. trachuri* was named by Iwamoto et al. (1995), but was deduced to be synonymous with *V. harveyi*, with the latter having precedence in taxonomy (Thompson et al. 2002). These workers carried out a detailed polyphasic taxonomy study employing phenotyping, 16S rRNA gene sequencing, fluorescent amplified fragment length polymorphisms (FAFLP), and DNA:DNA hybridization. The outcome was the realization that *V. trachuri* was highly related to *V. harveyi.*

Generally, *V. carchariae* and *V. trachuri* have been recovered from diseased fish, whereas *V. harveyi* was predominantly recovered from invertebrates. Therefore, the combined taxon is especially significant as a pathogen of marine vertebrates and invertebrates.

## The diseases

*Vibrio harveyi* has been linked with disease in a broad spectrum of warm water fish and invertebrates (Table 1). The list of hosts is extensive, and includes silvery black porgy (*Acanthopagrus cuvieri*) and brown spotted grouper (*Epinephelus tauvina*) in Kuwait (Saeed 1995), common dentex (*Dentex dentex*) and farmed sole (*Solea senegalensis*) in Spain (Company et al. 1999; Zorrilla et al. 2003), and farmed sea perch (*Lateolabrax japonicus*) and cage-reared grouper (*Epinephelus awoara*) in China (Qin et al. 2006; Wang et al. 2002). *V. harveyi* has been often associated with eye disease, for example in common snook (*Centropomus undecimalis*) (Kraxberger-Beatty et al. 1990), milkfish (*Chanos chanos*) in the Philippines (Ishimaru and Muroga 1997), and short sunfish (*Mola mola*) (Hispano et al. 1997). Without effective chemotherapy, the infected fish would inevitably become blind. In fish, the pathogen has also been attributed with gastro-enteritis, necrotizing enteritis, nodules on the opercula, scale drop and muscle necrosis, skin ulcers, tail rot, and vasculitis in different fish species (Table 1). With the latter infection, the fish were inappetant, lethargic, disoriented, and displayed necrotic subdermal cysts. Internally, meningitis, encephalitis, vasculitis, kidney necrosis, and liver and kidney damage were recorded (Grimes et al. 1985). Also, *V. harveyi* has been associated with a vibriosis-like disease in Japanese horse mackerel *(Trachurus japonicus)* when water temperatures were > 25 °C (Iwamoto et al. 1995). Here, fish displayed a diverse pathology, namely melanosis, erratic swimming behavior, bilateral exophthalmia, and hemorrhaging on/in the internal organs (Iwamoto et al. 1995). *V. harveyi* appears to exert great severity on/in immunosuppressed fish (Grimes et al. 1985).

**Table 1:** Diseases of marine vertebrates and invertebrates attributed to *Vibrio harveyi*

Disease	Host	Geographical range	Key references
Fish			
Eye disease	Common snook (*Centropomus undecimalis*)	USA	Kraxberger-Beatty et al. (1990)
	Milkfish (*Chanos chanos*)	Philippines	Ishimaru and Muroga (1997)
	Short sunfish (*Mola mola*)	Spain	Hispano et al. (1997)
Gastro-enteritis	Grouper (*Epinephelus coioides*)	Taiwan, China	Yii et al. (1997)
	Various fish, including black sea bream, Japanese sea bass, yellowfin sea bream, and red drum	Taiwan, China	Lee et al. (2002); Liu et al. (2003)
Necrotizing enteritis	Summer flounder (*Paralichthys dentatus*)	USA	Gauger et al. (2006); Soffientino et al. (1999)
Nodules on operculum	Tiger puffer (*Takifugu rubripes*)	Japan	Mohi et al. (2010)
Scale drop and muscle necrosis	Barramundi (*Lates calcarifer*)	Vietnam	Dong et al. (2017a)
	Hybrid grouper (*Epinephelus fuscoguttatus x E. lanceolatus*)	China	Zhu et al. (2018)
Skin ulcers	Shark (*Carcharhinus plumbeus*)	Italy	Bertone et al. (1996)
	Sole *(Solea senegalensis*)	Spain	Zorrilla et al. (2003)
	Hybrid grouper (*E. fuscoguttatus x E. lanceolatus*)	China	Shen et al. (2017)
Tail rot disease	Sea perch (*Lateolabrax japonicus*)	China	Wang et al. (2002)
	Sea bream (*Sparus aurata*)	Malta	Haldar et al. (2010)
Vasculitis	Brown shark (*Carcharhinus plumbeus*)	USA	Grimes et al. (1984)
Invertebrates			
Acute hepatopancreatic necrosis disease	Whiteleg shrimp (*Litopenaeus vannemei*)	Malaysia	Muthukrishnan et al. (2019)
Bacterial white tail disease	*Litopenaeus vannemei*	China	Zhou et al. (2012)
Black shell disease	Tiger shrimp (*Penaeus monodon*)	India	Selvin et al. (2005)
*Bolitas negricans*	Penaeid shrimp	Ecuador	Robertson et al. (1998)
Foot pustule disease	Abalone (*Haliotis discus hannai*)	China	Wang et al. (2018)
Luminous vibriosis	Penaeid shrimp	Ecuador, Asia	Prayitno and Latchford (1995)
Skin ulceration	Sea cucumber (*Holothuria scabra*)	Madagascar	Becket et al. (2004)
White patch disease	Seahorse (*Hippocampus kuda*)	India	Raj et al. (2010)
White spot on the foot	Japanese abalone (*Sulculus diversicolor*)	Japan	Nishimori et al. (1998)

An interesting situation occurred in Malaysia whereby farmed juvenile hybrid grouper (*E. polyphekadion × E. fuscoguttatus*) experienced losses of 29% in 10 days with diseased fish becoming lethargic, displaying excessive mucus production, fin rot, congestion of the brain, liver and kidneys, and splenic enlargement. *V. alginolyticus* and *V. harveyi* were cultured from diseased fish; both organisms exhibited pathogenicity in laboratory-based infectivity experiments. Indeed, use of both organisms administered concurrently led to more severe disease than when single cultures were used (Mohamad et al. 2019).

*Vibrio harveyi* has been particularly troublesome in shrimp culture. The pathogen has been recovered from outbreaks of *Bolitas negricans* (Spanish = small balls; diseased shrimps contain balled epidermal tissue which can block the digestive tract) in Ecuador (Robertson et al. 1998) and luminous vibriosis (the diseased animals can glow in the dark; Prayitno and Latchford 1995), and linked with black shell disease in tiger shrimp (*Penaeus monodon*) in India (Selvin et al. 2005) and bacterial white tail disease in whiteleg shrimp (*Litopenaeus vannamei*) in China (Zhou et al. 2012). With the latter disease, mass mortalities have been reported in which the affected shrimp displayed white or opaque lesions in the tail, which were attributed to muscle necrosis (Zhou et al. 2012).

The pathogen has been cultured from white patch disease in seahorses (*Hippocampus kuda)* (Raj et al. 2010). This disease, which was characterized by white patches on the surface and anorexia, resulted in high mortalities among the captive seahorses. Also, the pathogen has been associated with disease in lined seahorses (*Hippocampus erectus*) (Qin et al. 2017). Moreover, Chinese production of Japanese abalone (*Haliotis discus hannai*) experienced heavy mortalities attributed to *V. harveyi* (Nishimori et al. 1998)*.* The disease signs centered on the presence of white spots on the foot and eventually mortalities (Nishimori et al. 1998). Foot pustule disease was described in Japanese abalone, which were lethargic and weak, and exhibited pustles and atrophying of the foot muscle, leading to mortalities (Wang et al. 2018).

Luminous vibriosis caused by *V. harveyi* was reported in the packhorse rock lobster (*Jasus verreauxi*) larvae (Diggles et al. 2000). At the beginning of the infection, the larvae developed quickly expanding white spots, with death occurring within three days when the water temperatures were 20–23 °C, and the epidermis within the spots was completely destroyed (Diggles et al. 2000). Skin ulceration caused by *V. harveyi* was also documented in juvenile sea cucumber (*Holothuria scabra*) in Madagascar (Becket et al. 2004).

Together with *V. parahaemolyticus*, *V. owensii* (Xiao et al. 2017), *V. campbellii* (Dong et al. 2017b; Han et al. 2017), and *V. punensis* (Restrepo et al. 2018), *V. harveyi* has been reported to cause acute hepatopancreatic necrosis disease (AHPND) in whiteleg shrimp (*L. vannamei*) in Malaysian shrimp ponds. Moreover, pathogenicity of *V. harveyi* was confirmed in infectivity experiments (Muthukrishnan et al. 2019). This is interesting, because hitherto AHPND was associated exclusively with *V. parahaemolyticus.*

## Diagnosis

Confusion may have resulted previously from reliance on phenotypic diagnostic approaches when misidentification was unknown. Key diagnostic traits, which could allow confusion with *V. alginolyticus,* included the presence of fermentative motile Gram-negative rods that grew at 11–40 °C in 3–8% sodium chloride, produced catalase, oxidase, indole, and lysine and ornithine decarboxylase, but not arginine dihydrolase, reduced nitrates, was negative for the Voges Proskauer reaction, and decomposed blood, DNA, and gelatin but not aesculin, casein or starch, and utilized arabinose, cellobiose, glucose, and sucrose but not inositol or lactose (Austin and Austin 2016). However, modern molecular approaches have heightened the accuracy of diagnoses, and include 16S rRNA gene sequencing, which has become the favored method of identifying isolates accurately (Ransangan and Mustafa 2009) and multilocus sequence analysis, which successfully identified 36 isolates from Australia (Cano-Gomez et al. 2011). It is noteworthy that a polymerase chain reaction (PCR) based on the *V. harveyi toxR* gene was sensitive, i.e., capable of detecting 4.0 × 10^3^ cells/ml, and specific to *V. harveyi* but no other vibrios (Pang et al. 2006). However, a PCR was developed, which was based on the conserved sequence of the gene encoding the OMP *vhhP2*, and was shown to be quick and fairly specific, although some *V. campbellii* cultures cross reacted and were misidentified as *V. harveyi* (Cano-Gomez et al. 2011). Success has been reported for a multiplex PCR, which was both specific and sensitive (Pinto et al. 2017). Similarly, an enterobacterial repetitive intergenic consensus (ERIC)-PCR was developed and regarded as effective for detection of the pathogen (Xu et al. 2017). A highly sensitive [60 colony-forming units (CFU) per reaction] and specific recombinase polymerase amplification (RPA) was designed specifically to recognize the *V. harveyi toxR* gene. The researchers considered that the technique was fast and accurate, permitting effective monitoring of disease outbreaks (Pang et al. 2019).

Serological approaches have garnered support. For example, a chemiluminescent-based dot blot incorporating monoclonal antibodies was described, and invoked a brief 2-h incubation leading to a limit of detection of 2 × 10^5^/ml; this was regarded as 50 times more sensitive than the conventional dot blot, and was superior (~ 1000 times more sensitive) to the indirect enzyme-linked immunosorbent assay (Li et al. 2017).

## Pathogenic mechanisms

We will preface remarks by stating that much of the research on pathogenicity has involved use of laboratory cultures, some of which are of uncertain authenticity/identity. Therefore, there may be questions concerning whether or not the research has actually used genuine *V. harveyi* cultures, and the actual relevance of the data to the host. An initial conclusion may be reached that the pathogenicity mechanisms remain to be properly resolved. Nevertheless, infections and mortalities have been achieved using fish and cultures. For example, using *V. trachuri* and Japanese horse mackerel (*Trachurus japonicus*), which were maintained at a water temperature of 26 °C, 1.1 × 10^8^ cells/fish were injected intraperitoneally, and led to 100% mortalities within 24 h. However, using only 1.1 × 10^7^ cells/fish, a total of 50% mortalities occurred in four days. In comparison, immersion in 3.6 × 10^7^ cells/ml for 2 min led to 100% mortalities in three days. The disease signs matched those of naturally infected fish, namely erratic swimming and melanosis (Iwamoto et al. 1995). Yii et al. (1997) reported an LD_50_ dose for grouper (*Epinephelus coioides*) of 2.53 × 10^7^ CFU/g of fish. Moreover, they noted that the disease signs were similar to those of the natural infection, namely that the intestine was swollen and full of yellowish fluid. In comparison, Pujalte et al. (2003) reported an LD_50_ value of 1.5 × 10^5^ to 1.6 × 10^6^ CFU/fish for European sea bass (*Dicentrarchus labrax*). Similarly, Won and Park (2008) found the LD_50_ dose to Japanese flounder (*Paralichthys olivaceus*) and black rockfish (*Sebastes schlegeli*) to be 2.48 × 10^5^**–**8.76 × 10^7^ and 2.0 × 10^4^**–**2.52 × 10^6^ CFU/g of fish, respectively.

Therefore, how does the host respond to invasion by *V. harveyi*? Using suppression subtractive hybridization (SSH) technique, Wang et al. (2008) identified several immune-related genes, such as a heat shock protein (HSP) 70 gene and a major histocompatibility complex (MHC) class 1a gene, from kidney and spleen of turbot (*Scophthalmus maximus*) infected by *V. harveyi*. The rapid transcriptional upregulation after *V. harveyi* infection may be important for the survival of fish (Wang et al. 2008). Moreover, pathogenic *V. harveyi* isolates from diseased Japanese flounder were more resistant to the bactericidal effects of fish serum, and caused elevated respiratory burst activity in head kidney macrophages of the host. Apoptosis was induced in cultured fish cells, leading to apoptotic bodies, DNA fragmentation, and enhanced caspase-3 activity (Li et al. 2011).

Many virulence factors, such as hemolysins, proteases, lipopolysaccharide (LPS), the capacity to bind iron, interaction with bacteriophages, biofilm formation, and quorum sensing, have been identified (Table 2 and see below for details). It is certainly possible that pathogenicity reflects the interaction between two or more virulence factors functioning together or sequentially (Won and Park 2008). Moreover, it is important to realize that the pathogenicity mechanisms of *V. harveyi* to fish and invertebrates may well be different (Owens et al. 1996; Zhang 2001).

**Table 2:** Pathogenicity mechanisms

Pathogenicity mechanisms	Key references
Ability to bind iron	Owens et al. (1996)
Bacteriocin-like substance	Prasad et al. (2005)
Bacteriophage	Austin et al. (2003); Munro et al. (2003); Oakey and Owens (2000); Ruangpan et al. 1999
Biofilm—formation and attachment	Karasunagar et al. (1994)
Extracellular product—cysteine protease	Liu et al. (1996)
Extracellular product—lipopolysaccharide	Montero and Austin (1999)
Extracellular product—hemolysin	Bai et al. (2010); Deane et al. 2012; Sun et al. (2007); Zhang et al. (2001); Zhong et al. (2006)
Luminescence and quorum sensing	Henke and Bassler (2004); Nakayama et al. (2005); Yang et al. (2011)
Resuscitation from VBNC state	Sun et al. (2008)

### Ability to bind iron

It has been argued that the ability of pathogens to bind iron may be important for fish but not for invertebrates (Owens et al. 1996).

### Bacteriocin-like substances (BLIS)

BLIS was isolated from a culture, which was pathogenic to salmonids, i.e., *V. harveyi* VIB 571, and determined to be inhibitory to four other *V. harveyi* isolates and to other *Vibrio* spp., e.g., *V. parahaemolyticus*. BLIS was prepared from culture supernatants, and characterized as a unique protein with a molecular weight of ~ 32 kDa (Prasad et al. 2005).

### Bacteriophage

Bacteriophages have been associated with virulence (Austin et al. 2003; Oakey and Owens, 2000). In particular, *V. harveyi* myovirus like (VHML) phage is a temperate bacteriophage with a narrow host range that could enhance and even restore virulence of *V. harveyi* isolates to Atlantic salmon (*Salmo salar* L.) and increase hemolytic activity (Austin et al. 2003; Oakey et al. 2002). Infecting an avirulent *V. harveyi* strain VIB 642 with VMHL led to virulence and upregulation of hemolysin secretion (Munro et al. 2003). Knowledge of VMHL increased as the complete nucleotide sequence and the open-reading frame (ORF) were determined (Oakey et al. 2002). Furthermore, it has been suggested that bacteriophage may mediate toxicity of isolates in tiger shrimp by transfer of one or more toxin genes or genes controlling toxin production (Ruangpan et al. 1999). Subsequently, a novel siphovirus-like phage 1 (VHS1) was recovered, but a connection with virulence remains to be established (Khemayan et al. 2006; Pasharawipas et al. 2005).

### Biofilms

Biofilms form when organisms are attracted towards or arrive by accident at a surface, become attached, and then multiply. Specific attachment to chitin by means of chitin-binding proteins is an important mechanism for adhesion and colonization of *V. harveyi* (Montgomery and Kirchman 1993, 1994). Research has demonstrated that *V. harveyi* may survive in shrimp hatcheries due to the ability to form biofilms, which confer resistance to antibiotics and disinfectants (Karunasagar et al. 1994).

### Extracellular products (ECP)

*Vibrio harveyi* has been reported as slightly cytotoxic to fish and invertebrates, and produces ECP (Table 2; Liu et al. 1996; Won and Park 2008; Zhang and Austin 2000), which comprise hemolysins (Li et al. 2011; Zhang and Austin 2000), caseinase, gelatinase, lipase, and phospholipase (Liu et al. 1996; Zhang and Austin 2000). When brine shrimp (*Artemia franciscana*) nauplii were injected with a range of *V. harveyi* strains, correlations between mortality of brine shrimp and production of proteases, phospholipases, or siderophores of *V. harveyi* cultures were observed, but there is no correlation between mortality and hemolytic activity, hydrophobicity, or lipase or gelatinase production (Soto-Rodriguez et al. 2003). A 22 kDa extracellular cysteine protease has been purified (Fukasawa et al. 1988a, b; Liu et al. 1997) and considered to be the major exotoxin to tiger prawns (Lee 1996; Lee et al. 1997; Liu and Lee 1996). Moreover, Harries and Owens (1999) isolated two proteins, which caused mortalities in tiger prawns, and were regarded as both exotoxins and the probable virulence factors. When the ECP of pathogenic strain E2 was heated at 100 °C for 10 min or digested with proteinase K, it produced the same pathology in Dublin Bay prawns (*Nephrops norvegicus*) as crude, untreated ECP. Western blotting revealed the presence of low-molecular-weight LPS, which was considered to constitute the lethal toxin (Montero and Austin 1999). However, the same strain was not pathogenic to rainbow trout (*Oncorhynchus mykiss*) (Zhang 2001), indicating that the pathogenicity mechanisms of *V. harveyi* to shrimp and fish may well be different.

### Hemolysins in the ECP

**Fig. 2:**
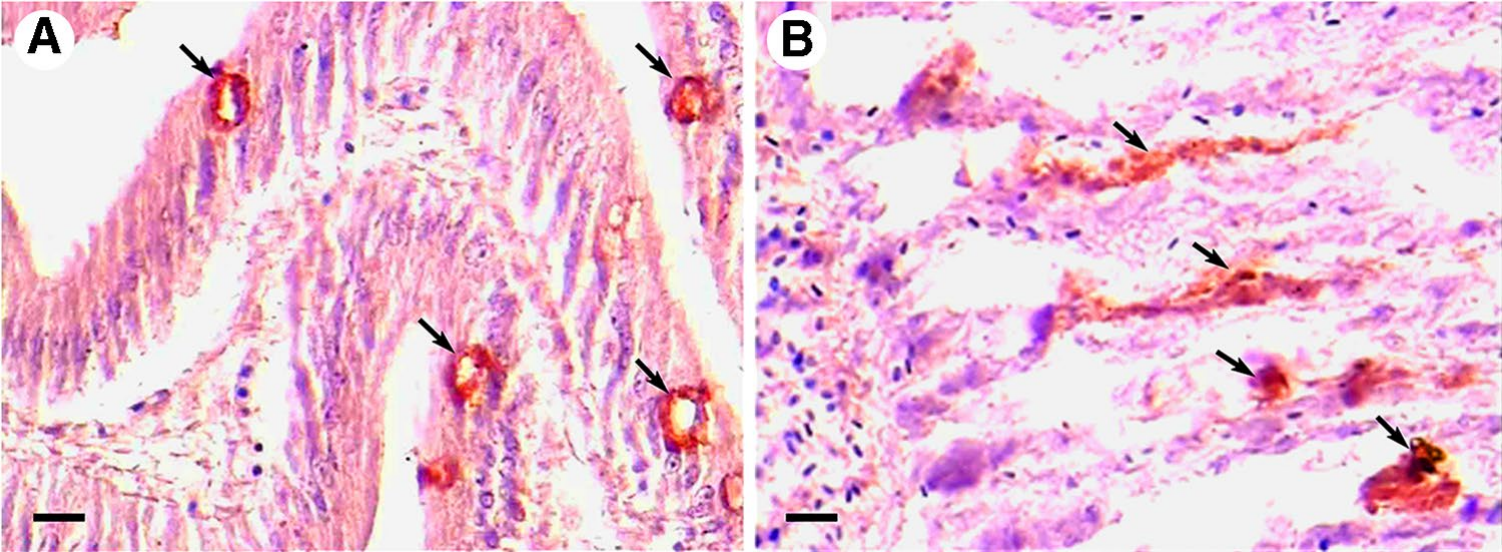
Immunohistochemical localization of recombinant VHH from *Vibrio harveyi* in tissues of Japanese flounder injected with VHH. Rabbit anti-VHH antibody was used as primary antibody and horseradish peroxidase (HRP)**-**labeled goat anti-rabbit antibody was used as the secondary antibody. Positive staining (arrows) in intestine (**a**) and gill (**b**). Bar = 20 µm

Zhang and Austin (2000) reported that the most highly pathogenic culture in their study was *V. harveyi* VIB 645, which produced ECPs with the highest titer of hemolytic activity to both Atlantic salmon and rainbow trout erythrocytes. They further correlated virulence of *V. harveyi* to salmonids with the possession of duplicate hemolysin genes, i.e., *vhhA* and *vhhB.* The majority (19/20) of the less virulent or avirulent cultures possessed single genes or none at all (Zhang et al. 2001). The open-reading frame (ORF) of the *vhhA* and *vhhB* genes was calculated to be 1257 nucleotides in length, which matches the *tlh* gene of *V. parahaemolyticus* (Nishibuchi and Kaper 1985). VHH hemolysin was determined to be homologous with lecithinase of *V. cholerae* and *V. mimicus*. The *vhhA* gene was overexpressed in *Escherichia coli,* and the purified recombinant VHH hemolysin was found to be cytotoxic to Japanese flounder gill cells. The protein caused mortalities in Japanese flounder with the LD_50_ dose calculated as 18.4 µg of protein/fish (Zhong et al. 2006). Sun et al. (2007) identified VHH hemolysin as a phospholipase B, and reported that a single residue change (Ser153) in the VHH hemolysin resulted in loss of hemolysin and phospholipase activities, and pathogenicity to turbot. In experiments involving immunolocalization of VHH in tissues of Japanese flounder challenged by the protein, the hemolysin was observed to be confined mainly to intestinal epithelial and gill epithelial cells (Fig. 2), indicating that the intestines and gills may be the primary targets for the invasion of *V. harveyi* in fish (unpublished data). In addition, recombinant VHH hemolysin induced various apoptotic features (such as apoptotic bodies, chromatin condensation, increased number of TUNEL-positive apoptotic cells, and caspase-3 activity) in Japanese flounder gill cell line. These data led to the conclusion that VHH inactivated these cells by apoptosis, via the caspase activation pathway (Bai et al. 2010). Moreover, VHH induced apoptosis in silver sea bream (*Sparus sarba*) and black sea bream (*Mylio macrocephalus*) fibroblast cell lines. This featured reduced mitochondrial membrane potential prior to increased caspase-3 activity. In short, it was highlighted that hemolysin led to cell death by induction of apoptosis (Deane et al. 2012).

### Luminescence and quorum sensing

Some *V. harveyi* strains possess luminescence characteristics when grown either in vitro or in vivo (Fig. 1b; Zhang 2001). Luminous vibriosis is so named, because diseased shrimp luminesce, i.e., glow in the dark (Prayitno and Latchford 1995). This raises the question concerning the role of luminescence in virulence. Luminescence may be verified by monitoring the expression of the *LuxR* gene in quorum sensing. Research has shown that the expression of LuxR and toxicity to shrimp was most important in relation to luminescence (Nakayama et al. 2005). *V. harveyi* had been confirmed to have all three types, i.e., AHL, AI-2, and CAI-1, of quorum-sensing activities (Yang et al. 2011). Additionally, quorum sensing regulates other virulent factors, such as type III secretion system (Henke and Bassler 2004).

### Resuscitation from the viable but nonculturable (VBNC) state

The VBNC state was first described by Xu et al. (1982), and referred to a state where bacteria remain viable and metabolically active, but are not culturable on standard bacteriological growth media. It is reasoned that the VBNC state is a survival strategy, which has been used by bacteria in response to harsh environmental conditions, and the bacterial cells in VBNC state can return to activity and culturability under appropriate circumstances (Oliver 2010). Sun et al. (2008) determined that *V. harveyi* could enter VBNC state, and the VBNC cells could be resuscitated via an increase in temperature when grown in the presence of yeast extract and Tween 20 or vitamin B. The resuscitation of dormant cells may well be one of the important reasons for vibriosis outbreaks in aquaculture.

## Disease control

The spread of disease associated with *V. harveyi* has inevitably led to a multiplicity of studies aimed at control. Interest started with the use of antimicrobial compounds of which Prefuran (Argent) administered at 0.1 mg/L for an unspecified period (Kraxberger-Beatty et al. 1990) and oxytetracycline (Saeed 1995) were successful. However, the concern over tissue residues and the development and spread of antibiotic resistance contributed to research in other areas of disease control, notably preventative rather curative.

### Bacteriophage therapy

Some research has focused on bacteriophage therapy to control luminous vibriosis in shrimp aquaculture (Choudhury et al. 2019). A lytic Myoviridae, VhKM4, was recovered (Lal et al. 2017). Moreover, a cocktail of three bacteriophages of the Myoviridae and Siphoviridae, which were isolated from shrimp farms in India, inhibited *V. harveyi*, led to greater survival of tiger shrimp larvae compared to controls (Stalin and Srinivasan 2017).

### Biological control

Surface-associated bacteria, i.e., *Exiguobacterium acetylicum,* were recovered on dilution plates from sea cucumber (*Stichopus badionotus*) and used successfully to control *V. harveyi* disease in brown-marbled grouper fingerlings (*Epinephelus fuscoguttatus*) (Alipiah et al. 2016). Rearing Pacific white shrimp (*Litopeanaeus vannamei*) in biofloc has been suggested to reduce hepatopancreas lesions attributed to *V. harveyi* (Aguilera-Rivera et al. 2017).

### Dietary supplements

A wide range of dietary supplements have been proposed for the control of *V. harveyi.* Some have been commercialized particularly in the Far East for use with fish and shellfish (e.g., Geng et al. 2012). Other examples for use in fish include β-1,3-glucan (Lee et al. 2018), bovine lactoferrin (Esmaeili et al. 2019), Chaga mushroom (Harikrishnan et al. 2012a), the seed of the evergreen tree *Leucaena leucocephala* (Verma et al. 2018), garlic (*Allium sativum*) (Talpur and Ikwanuddin 2012), ginger (*Zingiber officinale*), green tea (*Camellia sinensis*) (Harikrishnan et al. 2011b), hawthorn (*Crataegus* sp.) extract (Tan et al. 2017), Japanese pepper tree *(Zanthoxylum piperitum)* (Harikrishnan et al. 2012d), kudzu (*Pueraria thunbergiana*) (Harikrishnan et al. 2012b), loquat tree (*Eriobotrya japonica*) (Kim et al. 2011), and pericarp (*Zanthoxylum piperitum*) (Harikrishnan et al. 2012a). Immunomodulation and protection against challenge were common to these dietary supplements. For example, Chaga mushroom, when fed for 30 days as an ethanolic extract at 1.0% and 2.0% to kelp grouper (*Epinephelus bruneus*), was reported to achieve increased weight, immunomodulation, and protection against challenge with *V. harveyi* (Harikrishnan et al. 2012a).

Numerous dietary supplements have been researched for use with shrimp, and include black nightshade (*Solanum nigrum*) extract (Harikrishnan et al. 2011a) and mangrove (*Rhizophora apiculata*) leaf extract (Kannappan et al. 2018). Feeding 0.1% and 1.0% black nightshade, Harikrishnan et al. (2011a) reported significantly enhanced hemocyte count, glutathione peroxidase, phagocytic, phenoloxidase, respiratory burst and superoxide dismutase activities, and protection against infection with *V. harveyi.*

Probiotics have been evaluated for control of *V. harveyi* infections. For example, *Bacillus coagulans* achieved success when fed at 10^9^*-*cells/g of feed to the freshwater prawn, *Macrobrachium rosenbergii*. After 60 days, there was improved growth, significantly enhanced lysozyme and respiratory burst activities, and increased resistance to challenge (Gupta et al. 2016). Schaeck et al. (2016) were an advocate of *V. lentus,* which protected gnotobiotic (= germ-free) sea bass (*Dicentrarchus labrax*) larvae against challenge with *V. harveyi*. The benefits of feeding marine red yeast, *Rhodotorula mucilaginosa*, at 1.0 g/kg for eight weeks have been extolled (Zhou et al. 2016). In addition, it was found that a marine antagonistic strain *Pseudoalteromonas flavipulchra* JG1 could inhibit the growth of *V. harveyi* (Jin et al. 2010). From this culture, several small molecule compounds (p-hydroxybenzoic acid, trans-cinnamic acid, and 6-bromoindolyl-3-acetic acid) with inhibitory effect to *V. harveyi* were purified (Yu et al. 2012). This bacterial strain could be used as a potential probiotic to control *V. harveyi* disease.

A common theme with the dietary supplements is evidence for improved growth performance, immunomodulation notably of cellular and innate immunity, and protection against challenge with *V. harveyi.* This raises the question concerning the reason(s) for the profound benefit of what is essentially the addition a small quantity of dietary supplement. Could the reason reflect inadequacies in the formulation of diets or are these truly supplements, i.e., supplementing already adequate feed?

### Inhibition of quorum sensing

Quorum-sensing signal molecules may be involved in regulating virulence. In this connection, the 28 kDa AiiA protein of *Bacillus thuringiensis* interfered with quorum sensing in *V. harveyi*, and could be considered for involvement in disease control strategies (Bai et al. 2008).

### Vaccines

Although vaccines are primarily used to control diseases of fish, some commercial products containing *V. harveyi* antigens have been marketed for the control of shrimp diseases in the Far East. Nevertheless, most of the research has been directed at the control of fish disease.

There has been a dramatic improvement in the success of vaccines for the control of *V. harveyi* diseases reflecting some excellent science (Table 3). Efforts started with the traditional approach of inactivated whole-cell preparations before progression to consideration of purified subcellular components, and subsequently to the modern era of DNA vaccines.

**Table 3:** Methods of disease control

Method of disease control	Key reference(s)
Antimicrobial compound	Kraxberger-Beatty et al. (1990); Saeed (1995)
Bacteriophage therapy	Choudhury et al. (2019); Stalin and Srinivasan (2017)
Biological control	Aguilera-Rivera et al. (2017); Alipiah et al. (2016)
Dietary supplements—fish	Esmaeili et al. (2019); Lee et al. (2018)
Dietary supplements—invertebrates	Harikrishnan et al. (2011a); Kannappan et al. (2018)
Dietary supplements—probiotics	Gupta et al. (2016); Schaeck et al. (2016)
Inhibition of quorum sensing	Bai et al. (2008)
Vaccines—whole-cell vaccines	Crosbie and Nowak (2004); Harikrishnan et al. (2012b, c); Huang et al. (2012); Nguyen et al. (2017); Xu et al. (2019)
Vaccines—subunit vaccines	Atujona et al. (2019); Mao et al. (2011); Nguyen et al. (2018); Pang et al. (2010); Zhang et al. (2008); Zhu et al. (2019)
Vaccines—live vaccines	Cheng et al. (2010); Chin et al. (2020); Hu et al. (2011); Mohd-Aris et al. (2019); Sun et al. (2009)
Vaccines—DNA vaccines	Hu and Sun (2011); Wang et al. (2011, 2017)
Immunostimulation	Baletal and Gomez-Chiarri (2016)

*Whole-cell vaccines* In their simplest form, whole-cell vaccines comprise bacterial suspensions, which have been inactivated with chemicals, typically formalin. Whole-cell vaccines have gained considerable success with the control of some fish diseases, and numerous examples have been commercialized, worldwide. The approach has been used to control diseases attributed to *V. harveyi.* For example, a whole-cell vaccine administered by a variety of techniques, i.e., intraperitoneal injection, anal intubation, and, by immersion, to barramundi (*Lates calcarifer*) led to demonstrable antibody production. Clearly, the data revealed that barramundi could mount an immune response to vaccination (Crosbie and Nowak 2004). A bivalent vaccine comprising formalized cells of *V. harveyi* and *Photobacterium damselae* subsp. *piscicida* together with ECP was used in sole both involving immersion together with a booster dose or by i.p. injection. The outcome was excellent protection [relative percent survival (RPS = ~ 88%) for a 4-month period after vaccination]. Subsequently, protection declined (Arijo et al. 2005). A multivalent inactivated vaccine including cells of *V. alginolyticus*, *V. harveyi*, *V. vulnificus,* and infectious spleen and kidney necrosis virus was administered to groups of orange-spotted grouper (*Epinephelus coioides)* leading to excellent protection after challenge (RPS = 80%; Huang et al. 2012). The commercial attraction to inactivated whole vaccines is that they are comparatively inexpensive to produce. Consequently, research has been directed towards improving the performance of these vaccines, such as by entrapping the antigens in liposomes (Harikrishnan et al. 2012c). Use of CpG oligodeoxynucleotide 1668-enriched plasmid (p60CpG) as an adjuvant with formalized cells led to protection (RPS = 96.2%) in orange-spotted grouper (Nguyen et al. 2017). This led to immunomodulation and increased protection (Harikrishnan et al. 2012b). A similar positive effect resulted from the addition of adjuvant, Montanide, to formalin-inactivated cells, which were used to inject turbot. The vaccine was administered at 1.0 × 10^8^ cells/fish leading to an RPS of 75% after four weeks (Xu et al. 2019).

*Subunit vaccines.* Many articles have been published showing the success of subunit vaccines*.* ECPs, outer membrane proteins (OMP) (Arijo et al. 2008), and the hemolysin protein expressed in yeast (*Saccharomyces cerevisiae*) (Zhu et al. 2006) were immunogenic. Pang et al. (2010) described the use of OmpN mixed with Freunds complete adjuvant (FCA) to administer by i.p. injection (dose = 100 µl/fish) estuary cod (*Epinephelus coioides*) leading to an RPS or 60% or 70% depending on the challenge culture. A recombinant fusion protein r-OmpK-OmpU controlled disease in orange-spotted grouper (RPS = 81.8%) (Nguyen et al. 2018). Subsequently, the OMP To1C, which is a major adhesin, was used successfully to protect hybrid grouper (*E. fuscoguttatus* × *E. lanceolatus*) against experimental challenge (Zhu et al. 2019).

Molecular approaches to vaccine development have met with some success as illustrated by the study of Zhang et al. (2008), who cloned OMP genes, *OmpK* and *GADPH;* the recombinant proteins from which were expressed in the vector pET-30a(+). After purification of the protein, 100 µg quantities/fish were injected intraperitoneally into groups of yellow croaker (*Pseudosciaena crocea*). A booster dose was administered at three weeks. Use of r-OmpK and r-GADPH led to RPS values of 37.7% and 40%, respectively. Although some protection occurred, the levels were not as good as some of the studies using simpler whole-cell vaccines. In a subsequent development, expression of OmpK was achieved using the yeast *Pichia pastoris.* Experiments used groups of sea bass (*Lateolabrax japonicus*) and oral uptake of OmpK in alginate microspheres for five days. The outcome was an RPS of 61.5% (Mao et al. 2011). A recombinant protein vaccine, which was developed from the *VirB11* gene, was trialed in groups of orange-spotted grouper. After challenge, an RPS of 88% was achieved (Atujona et al. 2019).

*Live vaccines.* We have two concerns about live vaccines that need to be voiced. First, what is the possibility that the presence of live vaccines cells in and around a fish holding facility could be mistaken for the presence of a population of viable pathogens cells? The presence of such viable cells could confuse diagnostics. The second concern revolves around whether or not it is possible for an attenuated vaccine cell to acquire virulence determinants in the aquatic environment. There is not any evidence for these concerns, but it is considered necessary for scientists to be aware of and not ignore the issues. The attraction for live vaccines is that their use is more likely to mimic the infection process of viable pathogens. Therefore, the host responses are likely to be more natural and protective!

A live recombinant vaccine based on the OMP VhhP2 was injected intraperitoneally leading to excellent protection of Japanese flounder (RPS = 92.3%) (Sun et al. 2009). This was followed by a series of vaccination studies leading to commendable levels of protection. Thus, Cheng et al. (2010) used a recombinant protein, Vhp1, which when expressed in *Escherichia coli* and used as a live vaccine achieved an RPS of 90%. A live recombinant vaccine was developed by Hu et al. (2011), who expressed DegQ. The vaccine was injected intraperitoneally into turbot leading to an RPS of 90.9%. By oral and immersion, ROS values of 60.5% and 47.1%, were recorded, respectively. The vaccine was used by oral and immersion in a field trial leading to RPS values of 77.8% after a month and 81.8% after two months (Hu et al. 2011). A study by Mohd-Aris et al. (2019) involved a live protease-deletion mutant of *V. harveyi,* which was used to vaccinate grouper (*Epinephelus fuscoguttatus*) intraperitoneally with a dose equivalent to 10^5^ colony-forming units/fish. Following challenge after four weeks, an RPS of 52% was recorded (Mohd-Aris et al. 2019). A live-attenuated *V. harveyi* culture MVh_vhs (LAVh) was used in a 1-h bath with Asian seabass (*Lates calcarifer*) fingerlings leading to 68% survival after challenge (Chin et al. 2020).

*DNA vaccines.* A DNA vaccine, pcDNA-GPx, was developed by incorporating the glutathione peroxidase *GPx* gene into the pcDNA3.1 ( +) plasmid, which was injected intramuscularly into orange-spotted groupers leading after challenge to an RPS of 77.5% (Wang et al. 2017). Wang et al. (2011) achieved as RPS of 100% in turbot using the purified 35 kDa OMP OmpU at a 50 µg dose applied by intramuscular injection. A DNA vaccine in which the *ompU* gene was inserted into pEGFP-N1 plasmid was less successful when injected in 10 µg quantities intramuscularly in turbot. With this, the RPS was only 51.4%. DegQ and Vhp1 were incorporated into a DNA vaccine for use in Japanese flounder in which the resulting RPS was 84.6% (Hu and Sun 2011).

Although many publications have been encouraging in terms of success of vaccines after challenge, it remains to be determined which vaccines could be produced at the price that the users would be prepared to pay.

### Immunostimulation

Summer flounder (*Paralichthys dentatus*) were immersed or injected intraperitoneally with a hot water extract of brown seaweed (*Sargassum oligocystum*) leading to significant stimulation of respiratory burst and hematocrit, and less mortalities after challenge (*Sargassum oligocystum*) (Baletal and Gomez-Chiarri 2016).

## Conclusions

*Vibrio harveyi* is a serious pathogen for multiple species of marine fish and invertebrates particularly occurring in the warm waters of Asia, southern Europe, and South America. Some confusion has resulted with identification of cultures, particular centering on distinguishing *V. harveyi* and *V. campbellii*. However, taxonomy studies have confirmed the uniqueness of both of these species. Although virulence has been demonstrated with multiple isolates, there is a lack of clarity over the precise pathogenicity mechanisms, possibly reflecting differences between isolates as well as between fish and invertebrates, and overreliance on laboratory cultures, which are not necessarily reflective of the abilities of fresh isolates. Certainly, hemolysin has been the most extensively studied virulence factor of *V. harveyi* to date. Some excellent work has been accomplished, and it is realized that hemolysin is capable of killing fish cells by apoptosis via the caspase activation pathway. Maybe, key virulence genes, such as occur on plasmids and bacteriophages, become lost with storage of the so-called laboratory cultures. Fortunately, there has been much progress with the development of effective disease control strategies, including use of dietary supplements and vaccines. Clearly, further work is justified to better under this serious pathogen of marine animals.
